# Authors' Responses to Peer Review of “Offenders With Personality Disorder Who Fail to Progress: A Case-Control Study Using Partial Least Squares Structural Equation Modeling Path Analysis”

**DOI:** 10.2196/33933

**Published:** 2021-10-29

**Authors:** Georgina Mathlin, Mark Freestone, Celia Taylor, Jake Shaw

**Affiliations:** 1 Centre for Psychiatry and Mental Health Wolfson Institute of Population Health Queen Mary University of London London United Kingdom; 2 Alan Turing Institute London United Kingdom; 3 Millfields Unit John Howard Centre East London Foundation Trust London United Kingdom; 4 Oxleas NHS Foundation Trust London United Kingdom

*This is the authors’ response to peer-review reports for the paper “Offenders With Personality Disorder Who Fail to Progress: A Case-Control Study Using Partial Least Squares Structural Equation Modeling Path Analysis”.*

## Round 1 Review

### Overview of Changes

Several changes have been made to the manuscript [1] in response to the comments provided by both reviewers. These include additional information about personality disorder (PD) being included in the Introduction to further contextualize the population of interest in this paper. Information about the risk needs responsivity (RNR) model and readiness for treatment has been removed from the Introduction. Further details have been provided in the Methods section, and a visualization of the data analysis plan has been included as a second figure. Within the Discussion section, a new subsection focusing on the clinical and practical implications has been added. General sentence restructuring and grammatical errors have been amended throughout.

### Individual Responses

#### Anonymous

##### Major Comments

1. The Introduction is interesting to read, easy to follow, and well structured. I think the only thing that it is missing is some more background information on PDs. This could involve simply defining PD in a general sense and providing some information surrounding risks associated with them in community populations as well as their prevalence rates in offender populations (with citations). I would probably put this information at the start of the introduction, before going into more specific detail surrounding offenders with PD. Some language/grammatical improvements are also required throughout the introduction.
  *Response*: This has been addressed in the revised manuscript; two new paragraphs have been included at the start of the Introduction. Language and grammatical improvements have been made; although this was from a general read through and if there are any specific sentences that need rewriting, please identify them for further revisions.
2. The “Procedure” section in the Methods seems unnecessary as its own section. I would consider incorporating this information in the “Sample” section, potentially changing the heading to “Sample and Procedure.”
  *Response*: This has been addressed in the revised manuscript; the Procedure section has been combined with the Sample section.
3. In the Results section, I would suggest adding the test statistics and *P* values to Table 1, as this would make it very easy to identify where there were key differences.
  *Response*: This has been addressed in the manuscript, two new columns have been added to Table 1 to provide test statistics and *P* values. As there are two different types of test statistics used in the table (*t* test and chi-square), a footnote at the end of the table has been provided to explain this.
4. The numbers and interpretation of the results in Table 1 are somewhat difficult to follow. Percentagewise, a higher proportion of the nonprogression group is single compared to the control group, but the chi-square test results indicate that the control group is more likely to be single than the nonprogression group (presumably based on the actual N). I think this needs to be clarified/made consistent in the manuscript.
  *Response*: This has been addressed in the manuscript; only the percentages are now reported in Table 1 to make it easier to interpret. The supporting text has also been revised as there were correctly identified inconsistencies here that are now correct.
5. I think that it would be informative for the reader for the authors to merge all the supplementary descriptive results tables (Tables S1-S4) into one table and incorporate this into the main text in the Results section (rather than supplemental materials), also adding the test statistics to the tables.
  *Response*: Test statistics and *P* values have been added to the supplementary tables; however, we do not think merging the tables and entering them into the main text would be a good use of the reader’s time. This is summary information about the sample and did not inform the subsequent model.
6. The Discussion section was particularly interesting to read. Given the potential clinical impact and practical nature of this study; the only thing I think the Discussion is missing is a section on clinical/practical implications of the study (which was very briefly touched upon in the Conclusion). I think that this would really benefit the manuscript and be of interest to readers.
  *Response*: A section considering the clinical and practical implications of the study findings is now included prior to the Conclusion in the Discussion section.

##### Minor Comments

1. This is very minor, but the “Engagement With Treatment and Treatment Noncompletion” in the Introduction could probably just be “Engagement With Treatment,” as this covers treatment noncompletion.
  *Response*: This has been addressed in the revised manuscript.
2. I would avoid using the term “personality disordered” throughout the manuscript.
  *Response*: This has been addressed throughout the revised manuscript and adapted to “offenders with personality disorder” instead. A search for the phrase has ensured any occurrence of the phrase is now removed.
3. The semicolons in the first paragraph of the “Engagement With Treatment and Treatment Noncompletion” section should be colons (and in some other parts of the manuscript).
  *Response*: This has been addressed throughout the revised manuscript.
4. Some of the study objectives in the Introduction could be made somewhat more specific/precise (eg, explicitly stating “offenders with PD,” rather than just offenders, or offenders that have not progressed on the Offender Personality Disorder [OPD] pathway).
  *Response*: This has been addressed in the study objectives, specifically the first objective has been rephrased.
5. The capitalization of “N” within tables needs to be made consistent throughout.
  *Response*: This has been addressed throughout the manuscript and in all tables.
6. When referring to tables in the manuscript, the “t” should be capitalized throughout (eg, “Table 2”).
  *Response*: This has been addressed in the revised manuscript; all references to tables are now capitalized.
7. In the Results section, there may in fact be too much detail regarding the structural model assessment—for example, there is no need to explain what each of the parameters mean/represent.
  *Response*: This has been addressed in the revised manuscript; details on the standardized root mean square residual, acceptable loadings, and average variance extracted has been removed.
8. *P* values should be reported as exact numbers (ie, to 3 decimal points), and *P* values of *P*=.000 should be reported as *P*<.001.
  *Response*: As per the guidance provided by JMIR [3], the *P* values are expressed to two digits and three digits for *P*>.001.

#### Reviewer CJ [4]

##### Major Comments

However, I do not like the fact that many different models are mentioned in the Introduction and that these models are not mentioned further on. These parts could be shortened, and the paper would certainly benefit from this. I found the subdivision of the Introduction into many subunits disruptive as well. There is no common thread to the Introduction. Please try to create a better text flow.

*Response*: The section on the RNR and readiness for treatment has been removed with more contextualized information on PD provided instead. We hope this has helped with the text flow and reduced the number of subsections in the Introduction.

1. A distinction between the different PDs would be appropriate, as the risk for offending is not the same for all PDs.
  *Response*: This has been addressed in the revised manuscript at the start of the Introduction; differing PD types are discussed and defined.
2. Additionally, please give some more information about PDs in general.
  *Response*: This has been addressed in the revised manuscript at the start of the Introduction.
3. “The OPD pathway is informed from the “What Works?” literature [5], the RNR principles [6] and the Good Lives Model (GLM; [7]. However, the RNR model been criticised for not providing clear guidance for therapists for engaging offenders lacking in treatment readiness [8]. The responsivity principle of the RNR model may not currently be effectively implemented in the OPD pathway and contributing to the problem of offenders being referred but not accepted to numerous OPD services.” Incorporating these resources adds no value in my opinion since there is no further information about these models.
  *Response*: This has been addressed in the revised manuscript, and this section has been removed from the Introduction.
4. “Attitudes towards treatment”: Please specify possible outcomes in the description.
  *Response*: Additional information about the possible outcomes that were measured in attitudes toward treatment is provided in the revised manuscript.
5. A descriptive visual representation of the analysis plan would be helpful.
  *Response*: This has been developed and provided as Figure 2 in the manuscript.
6. Null hypothesis significance testing (NHST) needs hypotheses. If you use NHST (ie, *χ*^2^ and *t* test) in part 1 of the analysis, you need to formulate hypotheses since blind testing always leads to results.
  *Response*: The purpose of this analysis was to explore possible sources of sampling bias in the case-control matching procedure; therefore, it was by definition exploratory and inductive, and to suggest this was driven by hypotheses would be inaccurate. We would draw the editor’s attention to highly cited case-control work elsewhere (eg, [9]) as an indication of how this analysis is typically presented, noting that there is no reference to hypothesis testing.
7. No correction (such as Bonferroni) was made, despite multiple testing. This should be done. If one does not do this, it is fine because the main goal of the study was the model, but this needs to be addressed and reflected upon.
  *Response*: A sentence regarding a significance threshold has been provided at the start of the Results section to address this.
8. Methodology and results flow into each other. Please find a way to separate this better. I would also like to see not only the inner model described in the methodology but also the outer model (eg, the factors of psychopathology).
  *Response*: The comment regarding the methodology and results is too vague, and it is not clear what was wanted here, and therefore, no amendments have been made. The outer model has now been added to the supplementary materials and can be used to sense check—it is referred to in this way in the revised manuscript.
9. “The latent variables create the inner model and the variables were connected using clinical knowledge and theory (Figure 1).”
  Please specify “clinical knowledge” and “theory.” This is quite speculative. In addition, Figure 1 would benefit from a more specific description.
  *Response*: Further detail here has been added to expand upon the clinical knowledge and theory used. The Introduction to the paper is also regarded as the theory used.
10. “Second, the outer loadings within the SEM [structural equation modeling] model suggest that the single most influential factor was psychopathy or psychopathic disorder, which has long been acknowledged as a limiting factor for treatment and rehabilitation [10]. It could be argued that psychopathic offenders are not best served on a pathway that caters for offenders with personality disorder in the broader sense of the diagnosis as their needs are known to be different [11].”
  Do you have a suggestion for these individuals?
  *Response*: Additional information has been added to the manuscript regarding this that suggests a sustained focus on engagement and prosocial lifestyle changes, rather than on maladaptive personality traits.
11. Other limitations are that you specify a model a priori and that so many factors are used for such a complex phenomenon (with a quite limited sample).
  *Response*: It was not clear what was wanted here, so no amendments in the revised manuscript have been made in relation to this.
12. In addition, one must see very critically that psychosis and PS are combined. The fact must be discussed as this is problematic, having a massive influence on therapy and behavior.
  *Response*: It is not clear what “PS” is referring to.
13. Please consider dividing the Conclusions section into Future Perspectives and Conclusions sections, as this seems kind of inconsistent.
  *Response*: A section on clinical and practical implications has been added into the Discussion; the final paragraph has been moved above the Conclusions and called Future Perspectives, and then the Conclusions section summarizes the paper.

##### Minor Comments

1. Please consider changing the title of the paper by omitting “PLS-SEM [partial least squares structural equation modeling] Path Analysis,” which is too technical in my opinion (or maybe do not use the abbreviation).
  *Response*: We are happy to change the title of the paper if the editor feels it is necessary.
2. Please divide the sentence in the Results section of the Abstract into two sentences and thereby avoiding the semicolon.
  *Response*: Having two sentences does not make sense, but the sentence has been restructured.
3. Please avoid the semicolons in the second paragraph and check the overall structure of the sentences (and use hyphens if appropriate). Check for missed words, sentence structure, and punctuation in the paper.
  *Response*: This has been addressed throughout the revised manuscript.
4. Stay consistent when using abbreviations—do not alternate “PD” and “personality disorder.”
  *Response*: This has been addressed throughout the revised manuscript; all should now appear as personality disorder (abbreviation only in the short running title).
5. Please define “NHS” (National Health Service) before using the abbreviation.
  *Response*: This has been addressed in the revised manuscript.
6. I would like to read more about the “screening algorithm” and which PDs this algorithm screens for.
  *Response*: Further information about the screening algorithm has been provided in the Methods section.
7. “However, several of us are clinicians working within the London Pathways Partnership (LPP), a consortium of NHS trusts delivering services within the OPD pathway, are aware of several individuals that no OPD service, in prison or the NHS, is prepared to accept.” Reformulate this sentence since it is not easily comprehensible.
  *Response*: This sentence has been rephrased in the revised manuscript.
8. Omit % in the brackets in each row of the tables.
  *Response*: All % in the brackets have been removed from the tables.
9. “Although the relationship between problematic custodial behaviour and service refusal was not strong, the results still emphasise that services aiming to support these individuals need be able to receive men with patterns of such behaviour and contain and manage ongoing episodes, without this resulting in treatment termination.” Please rephrase this, as it is a quite complicated sentence.
  *Response*: This sentence has been rephrased in the revised manuscript.

## Abbreviations

GLM: Good Lives Model
LPP: London Pathways Partnership
NHS: National Health Service
NHST: null hypothesis significance testing
OPD: Offenders Personality Disorder
PD: personality disorder
PLS-SEM: partial least squares structural equation modeling
RNR: risk needs responsivity
SEM: structural equation modeling
